# Programmable saponin biosynthesis from gene networks to predictive biomanufacturing

**DOI:** 10.3389/fpls.2026.1800149

**Published:** 2026-03-26

**Authors:** Yuan Wang, Jiahong Chen, Zhonghong Liao, Li Yin, Fei Shi, Genhe He, Yonghui Liao

**Affiliations:** 1 Key Laboratory of Jiangxi Province for Functional Biology and Pollution Control in Red Soil Regions, School of Life Sciences, Jinggangshan University, Ji’an, China; 2 Dalian Practical Biotechnology Co. LTD., Dalian, Liaoning, China; 3 Shanghai Agrobiological Gene Center, Shanghai, China

**Keywords:** enzyme engineering, plant cell factories, predictive biomanufacturing, saponin biosynthesis, synthetic biology

## Abstract

Saponins are a structurally diverse plant glycosides with important ecological functions and broad pharmaceutical and industrial value. Recent advances have shifted saponin research from descriptive pathway elucidation toward predictive and programmable biomanufacturing. High-quality genome assemblies, integrated multi-omics profiling, and metabolic gene cluster analyses have clarified the enzymatic logic and regulatory architecture underlying saponin biosynthesis and structural diversification, enabling quantitative modeling of pathway flux and identification of key regulatory bottlenecks. Building on these foundations, synthetic biology tools, including CRISPR-based transcriptional modulation, synthetic promoters, and transcription factor rewiring, allow precise and programmable control of biosynthetic networks. In parallel, structure-guided enzyme engineering and AI-assisted protein design accelerate the optimization of cytochrome P450s and glycosyltransferases, improving catalytic efficiency and pathway robustness. These strategies are implemented across multiple production platforms, including engineered microbes, plant suspension cells, hairy roots, and adventitious root systems, enabling iterative optimization through Design-Build-Tes-Learn-cycles. Together, this review synthesizes recent conceptual and technological advances, positioning saponins as a model system that bridges gene networks, regulatory logic, and industrial biomanufacturing, and highlighting a generalizable framework for predictive design and scalable production of complex plant natural products.

## 1 Introduction

Saponins are a structurally diverse group of plant secondary metabolites with surfactant properties, widely distributed across the plant kingdom. They consist of a hydrophobic aglycone (sapogenin) conjugated to one or more hydrophilic sugar chains, enabling a broad spectrum of biological activities. Based on the aglycone structure, saponins are generally classified into triterpenoid saponins, derived from the 30-carbon precursor 2,3-oxidosqualene, and steroidal saponins, originating from the 27-carbon cholesterol (Moses et al., 2014). In plants, saponins function primarily as inducible chemical defenses, conferring resistance against pathogens, herbivores and abiotic stresses through antimicrobial, antifungal, and deterrent activities (Szakiel et al., 2011). Beyond their ecological significance, saponins display diverse pharmacological properties, including anti-inflammatory, anticancer, immunomodulatory, and cholesterol-lowering effects, rendering them valuable in pharmaceutical, functional food, and cosmetic industries (Ho et al., 2020). Given their significant biological and economic importance, saponin biosynthesis has become a major research focus in plant specialized metabolism. Early studies primarily aimed at elucidating biosynthetic pathways, discovering relevant enzymes, and classifying structures. Recently, however, the field has shifted toward synthesizing these findings, as reflected in comprehensive reviews detailing advances in pentacyclic triterpenoid biosynthesis and highlighting the enzymatic framework responsible for scaffold formation and diversification (Li et al., 2023).

The biosynthesis of saponins involves a complex network of enzymatic reactions. It begins with the cyclization of 2,3-oxidosqualene by oxidosqualene cyclases (OSCs) to form triterpene or steroidal backbones. Subsequent modifications are catalyzed by cytochrome P450 monooxygenases (P450s) and UDP-glycosyltransferases (UGTs) (Moses et al., 2013). Recent research has demonstrated that genes encoding these biosynthetic enzymes are often organized into metabolic gene clusters, enabling coordinated expression and efficient saponin production, as observed in the avenacin cluster in oats (*Avena strigosa*) and steroidal glycoalkaloid clusters in solanaceous plants (Moses et al., 2014). While the biosynthetic pathways are increasingly well-characterized, the regulatory frameworks controlling saponin production in response to developmental and environmental cues remain less elucidated. Recent genome resources have accelerated gene discovery. For example, a telomere-to-telomere assembly of *Panax ginseng* (Song et al., 2024) revealed extensive duplication of oxidosqualene cyclase (OSC), cytochrome P450 (CYP), and UDP-glycosyltransferase (UGT) gene families, explaining the remarkable structural diversity of ginsenosides. Multi-omics-based correlation analysis of metabolites and transcripts has become a standard approach for identifying candidate OSCs, CYPs, and UGTs in emerging medicinal species (Wan et al., 2024). In *Saponaria vaccaria*, combined methyl jasmonate (MeJA) elicitation, transcriptomics, and metabolomics enabled the functional validation of CYPs and UGTs critical for oleanane-type saponins (Chen et al., 2023). Similarly, *Saponaria officinalis* pathway reconstruction identified not only canonical UGTs but also a non-typical glycosylhydrolase family enzyme as essential for sugar-chain elongation (Jo et al., 2025). These findings underscore that saponin structural diversity emerges from lineage-specific gene expansions and unexpected enzyme recruitments.

Recent developments in synthetic biology and systems metabolic engineering are transforming saponin research from descriptive pathway elucidation toward programmable and predictive biomanufacturing. Genome-scale metabolic models, flux balance analysis, and AI-assisted enzyme mining now enable quantitative prediction of pathway bottlenecks and regulatory leverage points (Jang et al., 2022; Khan and Khan, 2026; Wang et al., 2025). These computational insights can be translated into programmable biological interventions through CRISPR-based transcriptional modulation, synthetic promoter engineering, transcription factor rewiring, and dynamic regulatory circuits, allowing fine-tuned control of biosynthetic flux (Lian et al., 2017). Iterative design–build–test–learn (DBTL) cycles further integrate experimental feedback into predictive modeling, progressively improving engineering precision and scalability (Lian et al., 2018).

Nevertheless, current predictive frameworks remain inherently probabilistic rather than deterministic. The complexity of plant metabolic networks, extensive enzyme promiscuity, subcellular compartmentalization, and physiological heterogeneity of plant cell cultures collectively constrain predictive accuracy. Consequently, empirical optimization remains indispensable, and robust biomanufacturing pipelines must integrate computational modeling with iterative experimental validation. Recognizing these constraints is essential for setting realistic expectations and guiding rational system design. This review focuses on regulatory and engineering strategies for translational biomanufacturing, synthesizing advances in genomics, network analysis, synthetic biology, and bioprocess development. By tracing saponin research from pathway discovery to predictive engineering and industrial application, we highlight areas amenable to rational design versus those still requiring empirical input. Ultimately, we frame saponins as a model for how integrated systems and synthetic biology can advance next-generation natural product manufacturing.

## 2 The synthesis, transport, and transformation of saponins

The synthesis, intracellular transport, and metabolic transformation of saponins are tightly regulated processes that contribute to their structural diversity and functional versatility in plants. Saponin biosynthesis originates primarily from the cytosolic mevalonate (MVA) pathway, which generates the universal isoprenoid precursors isopentenyl diphosphate (IPP) and dimethylallyl diphosphate (DMAPP) (Thimmappa et al., 2014). These precursors are condensed by farnesyl diphosphate synthase to form farnesyl diphosphate (FPP), a 15-carbon intermediate. Two FPP molecules are then converted to squalene by squalene synthase, followed by oxidation to 2,3-oxidosqualene by squalene epoxidase (Moses et al., 2014). As a key metabolic branch point, 2,3-oxidosqualene is cyclized by distinct oxidosqualene cyclases (OSCs) to generate diverse triterpenoid and steroidal scaffolds. For example, β-amyrin synthase produces β-amyrin, the common precursor of numerous oleanane-type saponins, whereas cycloartenol synthase directs flux toward primary sterol biosynthesis (Haralampidis et al., 2002). Subsequent structural diversification is achieved through the sequential actions of cytochrome P450 monooxygenases (P450s) and UDP-glycosyltransferases (UGTs), which introduce oxygenated functional groups and sugar moieties, respectively (Seki et al., 2015). In *Medicago truncatula*, the P450 enzyme CYP716A12 has been characterized as a multifunctional oxidase critical for hemolytic saponin production (Carelli et al., 2011). Similarly, coordinated P450 and UGT activities underlie glycyrrhizin biosynthesis in Glycyrrhiza uralensis, illustrating the modular organization of saponin pathways (Moses et al., 2014). Comparative genomic and biochemical analyses across *Panax* species further reveal how enzyme family expansion and neofunctionalization drive the diversification of dammarane-type saponins, thereby establishing direct links between enzyme evolution and phytochemical diversity (Yun et al., 2024).

Although saponin biosynthesis occurs predominantly in the cytosol and endoplasmic reticulum, the final products are typically sequestered in the vacuole, a strategy that mitigates cytotoxicity and enables stable accumulation (Yazaki, 2006). However, the molecular mechanisms governing saponin transport remain incompletely understood. In *Glycyrrhiza glabra*, the triterpenoid saponin glycyrrhizin is transported via a two-step process. Proton-coupled symporters on the plasma membrane mediate glycyrrhizin uptake into the cytosol, driven by a proton gradient. Subsequently, ATP-binding cassette (ABC) transporters, particularly ABCC-type proteins, facilitate its MgATP-dependent transport across the tonoplast into the vacuole (Kato et al., 2022). While this model provides a valuable conceptual framework, its general applicability across saponin-producing species has yet to be systematically validated.

Beyond biosynthesis and sequestration, saponins also undergo extensive metabolic transformation, primarily through glycosylation and deglycosylation, processes that profoundly influence their bioactivity, solubility, and ecological function. For example, six UGTs (UGTPn17, UGTPn42, UGTPn35, UGTPn87, UGTPn19, and UGTPn12) from *Panax notoginseng* were identified. These enzymes are responsible for the efficient and direct enzymatic biotransformation of 21 triterpenoid saponins via 26 various glycosylation reactions (Li et al., 2022). Conversely, deglycosylation mediated by endogenous glycoside hydrolases or microbial enzymes removes sugar moieties, thereby increasing the lipophilicity of saponins and potentially enhancing their biological activity. This process is particularly well characterized in Panax ginseng, where intestinal microbes or lactic acid bacteria convert major ginsenosides such as Rb1 into compound K through sequential β-glucosidase-mediated hydrolysis, markedly improving gastrointestinal absorption and pharmacological efficacy (He et al., 2019).

Recent technological advances are accelerating the systematic decoding of saponin glycosylation logic. Substrate-multiplexed profiling platforms now enable high-throughput mapping of UGT donor–acceptor specificity, providing scalable tools to resolve complex glycosylation networks (Hao et al., 2025). Complementary studies have identified specialized enzymes, such as a 4′-O-rhamnosyltransferase acting on steroidal sapogenins, highlighting how discrete sugar decorations can fine-tune biological activity (Zhao et al., 2025). Collectively, these findings underscore that saponin diversity is not solely encoded at the level of scaffold biosynthesis, but is dynamically shaped by transport processes and post-synthetic transformations. Such modularity provides both evolutionary flexibility and a powerful entry point for metabolic engineering and biotechnological exploitation (Morshed et al., 2024).

## 3 Saponins as standard compounds and bioactives in Chinese herbal medicine

Saponins represent a major class of bioactive secondary metabolites widely distributed across the plant kingdom. In Central Asia alone, saponin-containing species have been reported in approximately 1,700 plants spanning 104 families, with pronounced enrichment in Araliaceae, Liliaceae, Fabaceae, and Dioscoreaceae, where substantial structural and functional diversification has been documented (Rai et al., 2021).

**Figure 1:**
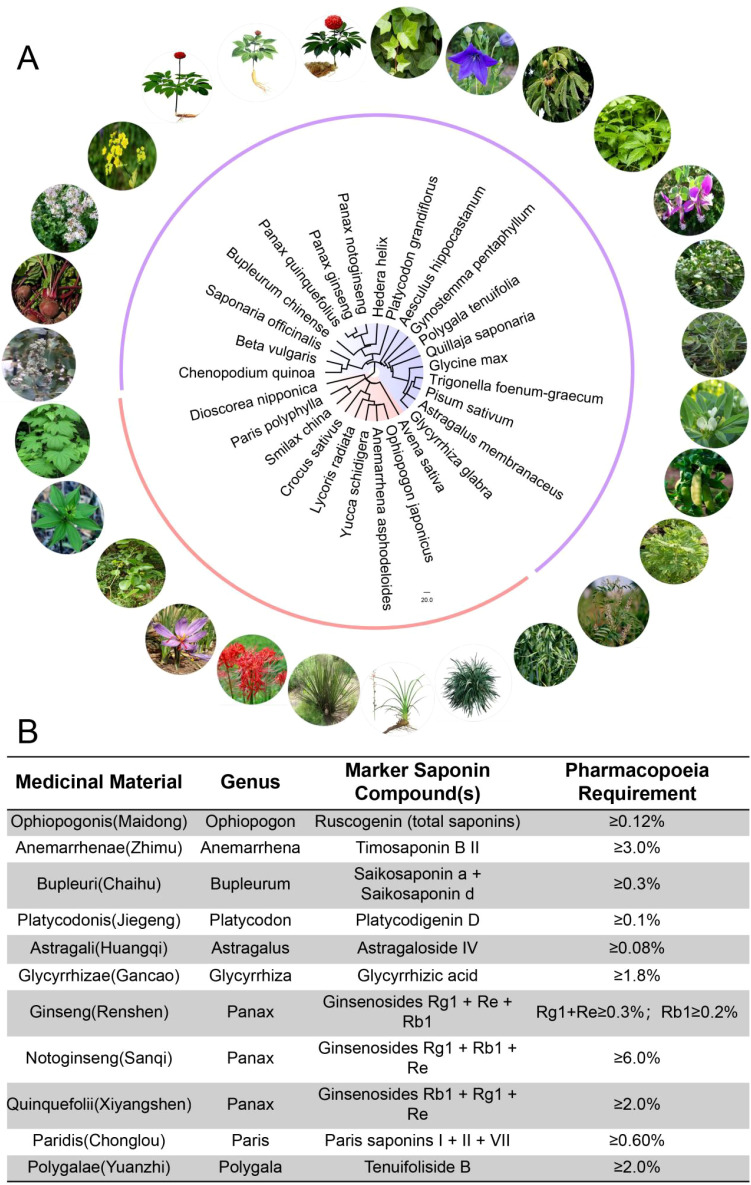
Phylogenetic distribution and phytochemical standards of key saponin-containing medicinal materials from the Chinese Pharmacopoeia (2025). **(A)** Circular phylogenetic tree illustrating the taxonomic relationships among the source plants, with corresponding morphological images. **(B)** Data table listing the ten medicinal materials, their taxonomic classification, marker saponin compounds, and the minimum content requirements as per the pharmacopoeia. The integrated visualization highlights the relationship between taxonomy and chemical markers for quality control.

Reflecting their established therapeutic relevance, the Chinese Pharmacopoeia (National Pharmacopoeia Committee, 2025) designates specific saponins as quantitative quality markers for a range of medicinal plants (**Figure 1**), thereby linking chemical composition to clinical efficacy and industrial standardization. Representative examples include steroidal saponins such as ruscogenins in *Ophiopogon japonicus* and timosaponins in *Anemarrhena asphodeloides*, triterpenoid saponins including saikosaponins in *Bupleurum* spp. and platycodon saponins in *Platycodon grandiflorus*, as well as cycloartane- and oleanane-type saponins such as astragaloside IV (*Astragalus membranaceus*) and glycyrrhizic acid (*Glycyrrhiza glabra*) (Lee et al., 2013; Moses et al., 2014; Xia et al., 2023). Within Araliaceae, *Panax* species are standardized by characteristic ginsenoside profiles that underpin immunomodulatory, neuroprotective, and cerebrovascular activities (Jang et al., 2023). Additional pharmacopoeial examples include anticancer polyphyllins from *Paris polyphylla* and neuroactive saponins from *Polygala tenuifolia* (Wang et al., 2024a). Together, these standards ensure batch-to-batch consistency and provide a regulatory foundation for clinical and commercial use.

Despite their established clinical value, sustainable and scalable supply of high-quality saponins remains a critical bottleneck, particularly for slow-growing or overharvested medicinal plants such as *Paris polyphylla*. Overreliance on wild resources has led to ecological pressure, supply instability, and significant fluctuations in active compound content. In response, recent advances in synthetic biology and metabolic engineering have enabled the reconstruction of complete saponin biosynthetic pathways in microbial hosts. Notably, the full biosynthetic pathway of the vaccine adjuvant QS-21 from *Quillaja saponaria* has been successfully reconstituted in yeast, providing a robust platform for scalable and controllable production (Liu et al., 2024). Parallel identification of key acyltransferases shaping QS-21 structure–activity relationships further illustrate how pathway elucidation can directly inform industrial vaccine adjuvant development (Chen et al., 2024). Together, these examples highlight the translational bridge between pharmacopoeial standardization, biosynthetic understanding, and scalable biomanufacturing.

## 4 Regulatory insights of saponin metabolism in plants

**Figure 2:**
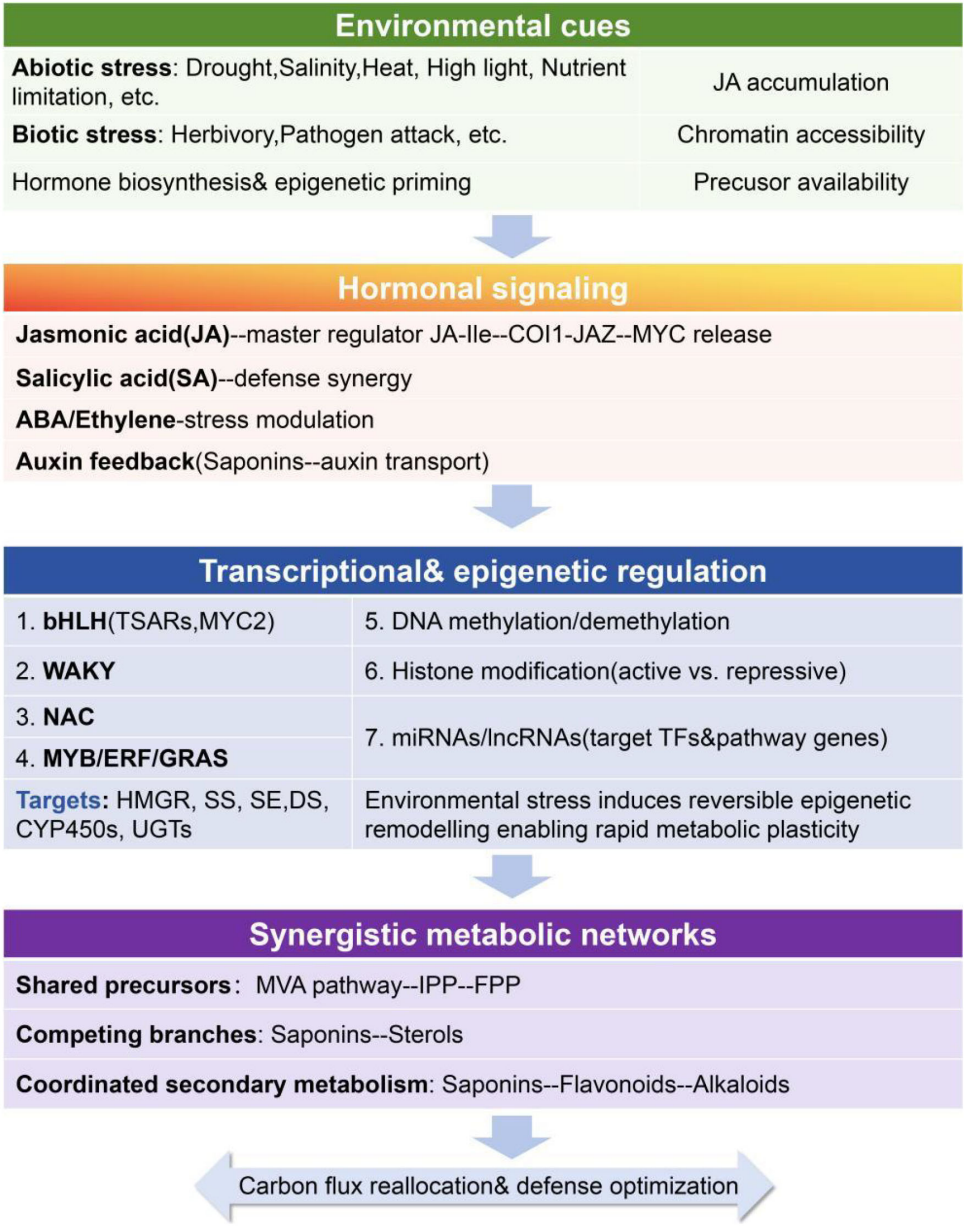
Multilayered regulatory framework governing saponin metabolism in plants. Environmental cues initiate hormonal signaling cascades dominated by jasmonic acid, which converge on transcriptional and epigenetic regulatory layers to fine-tune the expression of saponin biosynthetic genes. These regulatory outputs are further integrated with other secondary metabolic pathways through shared precursors, transcription factors, and regulatory RNAs, enabling dynamic allocation of metabolic resources in response to developmental and environmental demands.

The control of saponin biosynthesis operates through a multi-tiered regulatory system, encompassing responses to external environmental cues, chromatin-level modifications, and integrated metabolic networks. As summarized in **Figure 2**, these interconnected modules constitute an integrated regulatory framework that links upstream signal perception with downstream metabolic outputs, thereby shaping saponin content and structural diversity across tissues and developmental stages. At the systems level, saponin metabolism is embedded within broader regulatory circuits coordinating carbon allocation, redox homeostasis, and stress adaptation, enabling plants to translate diverse internal and external signals into coherent transcriptional and metabolic responses. Elucidation of this regulatory architecture is therefore fundamental for understanding plant adaptive strategies and for guiding the rational design of predictive metabolic engineering platforms.

### 4.1 Environmental influences on saponin metabolism

Environmental factors act as primary upstream drivers shaping saponin metabolism by modulating hormonal signaling, epigenetic states, and carbon allocation. Rather than responding passively, plants actively recalibrate saponin biosynthesis in accordance with stress type, intensity, and developmental context, reflecting the role of saponins as inducible chemical defenses rather than constitutive metabolites. For example, moderate drought in *Eleutherococcus senticosus* causes genome-wide DNA hypomethylation at saponin synthesis gene promoters, enabling increased saponin synthesis that enhances drought tolerance (Wang et al.,2024b). Similarly, moderate water deficit during flowering in *Panax notoginseng* increases total saponin content by 30–50%, accompanied by elevated expression of squalene synthase and downstream pathway enzymes, indicating transcriptional activation of flux-controlling nodes (Liao et al., 2017). Hormonal and metabolic signaling further integrate environmental information into pathway regulation. Foliar application of 5 mM leucine alleviates heat stress in *P. notoginseng* and restored photosynthetic efficiency, concomitantly elevating ginsenoside levels by ~40% (total PNS) versus untreated controls (H. Liu et al., 2023a). Salinity stress similarly enhances saponin accumulation in multiple species, including milk thistle and soybean, reflecting conserved stress-responsive regulatory modules that activate secondary metabolic pathways under osmotic and ionic perturbation (Zahra et al., 2022). In contrast, heavy-metal stress (e.g. cadmium, copper) can suppress saponin and flavonoid levels, indicating negative effects on plant metabolic profiles (Rabeh et al., 2025). Light availability represents another dominant environmental regulator. Elevated irradiance (400 vs. 100 μmol m^-^² s^-^¹) significantly promotes Paris saponin VII accumulation in *Paris polyphylla*, primarily by increasing precursor supply and intracellular compartmentalization efficiency (Wen et al., 2023). As a defense response to biotic stresses like herbivory and pathogen infection, a wide range of plants increase saponin production, with these molecules often functioning as phytoalexins (Moses et al., 2014). Collectively, these findings reveal that environmental regulation of saponin metabolism operates through an integrated control network encompassing epigenetic remodeling, hormonal signaling, metabolic flux redistribution, and stress-responsive transcriptional circuits. Rather than linear stimulus–response chains, these modules function as interconnected regulatory hubs that translate diverse external cues into coordinated metabolic outputs.

### 4.2 Hormonal and signaling pathways in saponin biosynthesis

Transcriptional regulation serves as a convergence point integrating environmental and hormonal signals to fine-tune saponin metabolism through hierarchical transcriptional networks. Jasmonic acid (JA) and salicylic acid (SA) are primary hormonal drivers that integrate stress signals into the saponin pathway, while abscisic acid (ABA) and ethylene provide essential modulation (Li et al., 2019). Rather than functioning as isolated pathways, these hormonal networks exhibit extensive cross-talk, collectively forming a multilayered regulatory system that orchestrates saponin biosynthesis as a core component of plant defense and stress adaptation. Jasmonic acid (JA) is the primary driver of inducible saponin biosynthesis across diverse plant lineages. Perception of JA initiates a conserved signaling cascade that culminates in the activation of core transcriptional regulators. In the absence of JA, JASMONATE ZIM-DOMAIN (JAZ) proteins repress downstream transcription factors. Upon JA accumulation, the bioactive conjugate jasmonoyl-isoleucine (JA–Ile) binds to the Skp1–Cullin1–F-box CORONATINE INSENSITIVE 1 (SCF^COI1) receptor complex, triggering ubiquitination and proteasomal degradation of JAZ repressors. This releases MYC-type basic helix–loop–helix (bHLH) transcription factors, which directly activate genes involved in triterpenoid backbone formation, oxidative tailoring, and glycosylation (Afrin et al., 2015). In *Calendula officinalis* hairy root cultures, JA treatment resulted in an approximately 86-fold increase in triterpenoid saponin levels (Rogowska et al., 2022). In *Panax* plants, JA activates MYB2, WRKY8/9 and other transcription factors (TFs) to drive triterpene saponin accumulation (Shen et al., 2025). SA also functions as a potent elicitor. As demonstrated in *Psammosilene tunicoides*, a one-day exposure to 5 mg/L SA increased the total saponin content by approximately 2.5-fold. Transcriptomic profiling revealed concomitant induction of genes encoding 1-deoxy-D-xylulose-5-phosphate synthase (DXS), squalene epoxidase (SE), and multiple transcription factors belonging to the WRKY and NAC families, indicating coordinated transcriptional activation of pathway flux ​ (Su et al., 2021). ABA also modulates saponin metabolism, particularly under osmotic stress conditions. For instance, in *Calendula* hairy roots, ABA treatment nearly doubled the accumulation of free oleanolic acid (the aglycone precursor) and enhanced the release of its saponin glycosides into the culture medium (Markowski et al., 2022). Ethylene exerts more nuanced regulatory effects. Although its direct influence on saponin accumulation is modest, ethylene strongly reshapes sterol metabolism, thereby indirectly influencing precursor availability and metabolic competition within the triterpenoid network (Markowski et al., 2022). Auxin does not act as a primary inducer of saponin biosynthesis. Instead, emerging evidence suggests reciprocal regulation, whereby certain steroidal saponins influence auxin transport and distribution. In *Arabidopsis thaliana*, the steroidal saponin protodioscin disrupts polar auxin transport, thereby modifying root architecture and developmental patterning (Santos Wagner et al., 2021).

At the signaling level, JA biosynthesis and perception provide a mechanistic framework linking environmental stress to saponin production. JA is synthesized from α-linolenic acid through the octadecanoid pathway, beginning with the action of lipoxygenase (LOX) to form 13-hydroperoxyoctadecatrienoic acid. This intermediate is converted by allene oxide synthase (AOS) and allene oxide cyclase (AOC) into 12-oxo-phytodienoic acid (OPDA), which is reduced and β-oxidized to produce JA (Wasternack and Hause, 2013). Conjugation with isoleucine by JAR1 generates jasmonoyl-isoleucine (JA–Ile), the bioactive signal perceived by the Skip1-Cullin1-F-box protein complex with COI1 (SCF^COI1) receptor. Binding of JA–Ile promotes the ubiquitination and degradation of JAZ repressors, thereby releasing MYC transcription factors to activate downstream defense and metabolic genes (Staswick and Tiryaki, 2004).

In *Panax* species, JA and its methylated derivative methyl jasmonate (MeJA) strongly activate genes encoding enzymes of the mevalonate pathway, including 3-hydroxy-3-methylglutaryl-CoA reductase (HMGR), farnesyl diphosphate synthase (FPS), and dammarenediol synthase (DDS), as well as downstream cytochrome P450 monooxygenases and UDP-glycosyltransferases, leading to marked enhancement of ginsenoside accumulation (Han et al., 2011). Transcriptome analyses further show that JA-responsive transcription factors, particularly WRKY, bHLH, and MYB families, act as direct regulators linking JA signaling to the activation of ginsenoside biosynthetic genes (Cao et al., 2015).

**Figure 3:**
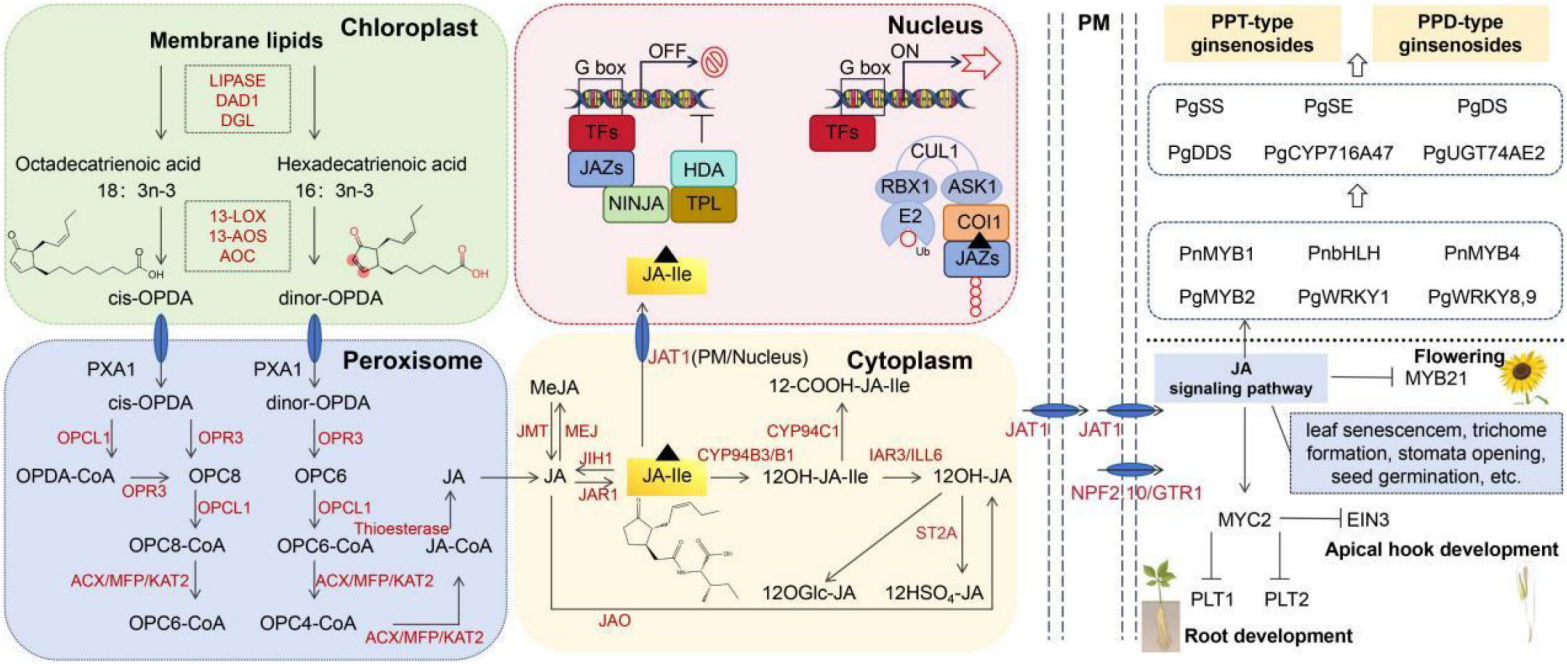
The jasmonic acid (JA) biosynthesis and signaling pathway and its regulatory role in plant development and saponin synthesis. This schematic diagram illustrates the multi-step JA biosynthetic pathway within chloroplasts and peroxisomes, and the subsequent JA signaling cascade in the nucleus. The pathway is organized according to subcellular localization (chloroplast, peroxisome, cytosol, nucleus), with key enzymes, intermediates, and core signaling components (e.g., JAZ repressors, MYC transcription factors) highlighted.

Owing to this strong inductive effect, MeJA is widely used as an elicitor in ginseng adventitious root and suspension cultures, where it consistently boosts ginsenoside yield (Cao et al., 2015). Beyond simple elicitation, manipulation of JA signaling components (e.g., JAZ repressors or MYC activators) is emerging as a promising strategy to enhance and fine-tune saponin accumulation (Lu and Hyun, 2021). Thus, JA functions not only as a stress-responsive hormone but also as a key metabolic switch that integrates environmental cues with the specialized production of pharmacologically valuable ginsenosides. Future work should focus on dissecting JAZ–MYC complexes and their cross-regulation with epigenetic modifiers, which may provide entry points for dynamic control of saponin flux. The JA biosynthetic and signaling cascade that connects environmental stress to saponin biosynthesis is illustrated in **Figure 3**.

### 4.3 Transcriptional and epigenetic control of saponin biosynthesis

The regulation of saponin metabolism by hormone signaling operates primarily through transcriptional and epigenetic layers, rather than direct enzymatic control. These regulatory layers determine the spatial, temporal, and quantitative expression patterns of biosynthetic genes, thereby shaping metabolic flux, tissue specificity, and stress responsiveness. JA–centered signaling cascades, together with auxiliary inputs from SA, ABA, and ethylene, are decoded by hierarchically organized transcription factor networks and chromatin-based mechanisms. Collectively, they translate transient hormonal signals into sustained, tissue-specific, and stress-adaptive transcriptional programs, thereby stabilizing saponin production under fluctuating environmental conditions.

Recent studies have revealed a conserved yet species-adapted regulatory architecture dominated by three major transcription factor (TF) families, namely basic helix–loop–helix (bHLH), NAM/ATAF/CUC (NAC), and WRKY proteins. Among them, bHLH transcription factors act as central integrators of jasmonate signaling. In *Medicago truncatula*, ACTIVATING REGULATORS 1 and 2 (TSAR1 and TSAR2) directly transactivate the β-amyrin synthase (β-AS), HMGR, and CYP93E2 promoters via G-box binding, coordinating the production of non-hemolytic triterpenes in response to JA (Mertens et al., 2016). This regulatory axis is antagonized by MtbZIP17, which disrupts TSAR1/2 transactivation under endoplasmic reticulum stress, effectively downregulating saponin biosynthesis (Ribeiro et al., 2022). The NAC transcription factors represent another key regulatory tier, particularly in stress-responsive reprogramming of saponin metabolism. In *Panax ginseng*, JA and cold stress induce saponin biosynthesis through the transcription factors PgNAC41–2 and PgNAC72, which mediate the coordinated activation of a set of core genes, including *HMGR*, squalene synthase (*SS*), squalene epoxidase (*SE*), dammarenediol synthase (*DS*), and *CYP716A47* (C. Liu et al., 2023a). The family of WRKY transcription factors act as key nodes linking biotic and abiotic cues to the regulation of saponin biosynthetic pathways. In *Panax ginseng*, several structurally distinct WRKY regulators have been functionally characterized. The transcription factors, PgWRKY1 and PgWRKY3 primarily influence downstream modification steps by modulating the expression of CYP716A47 and specific UDP-glycosyltransferases (Nuruzzaman et al., 2016), whereas PgWRKY4X exhibits a more stimulus-specific role, being induced by *Chaetomium globosum* challenge and selectively enhancing squalene epoxidase (SE) expression (Yao et al., 2020). In integrating JA and environmental signals, PgWRKY8 upregulates multiple pathway genes, such as HMGR, FPS, DS, and CYP716A53v2 (Xiu et al., 2016). Furthermore, this mechanism is conserved in *Panax quinquefolius*, where PqWRKY1 activates biosynthesis under JA elicitation (Sun et al., 2013). Specialized transcription factor families contribute distinct regulatory layers to saponin biosynthesis. PgMYB2 and PnMYB2/PnMYB61, for instance, fine-tune glycosylation and oxidation steps, while ethylene-responsive PgERF120 and JA-inducible PnERF1 optimize precursor flux (Deng et al., 2017; Man et al., 2023; Xia et al., 2022). Furthermore, PgGRAS68–01 gates gibberellin-mediated pathway induction, and the trihelix protein PgGT25–04 imposes ABA-dependent repression on early-pathway genes (Chang Liu et al., 2023a; Zhu et al., 2023). Within this tiered regulatory architecture, bHLH TFs act as central processors, NAC/WRKY TFs integrate environmental signals, and specialized TFs such as those described above function as precise modulators. This integrated system allows plants to dynamically balance saponin production with growth demands. Representative TFs and their corresponding target genes regulating saponin biosynthesis are summarized in **Table 1**.

**Table 1:** Transcription factors (TFs) that regulate the saponin biosynthesis.

TFs	Types	Species	Targets	Function	Reference
MtTSAR1	bHLH	*Medicago truncatula*	β-AS, HMGR, CYP93E2	Positive	(Mertens et al., 2016)
MtTSAR2	bHLH	*Medicago truncatula*		Positive	
MtbZIP17	bZIP	*Medicago truncatula*	SAR1 and TSAR2	Negative	(Ribeiro et al., 2022)
GubHLH3	bHLH	*Glycyrrhiza uralensis*	Soyasaponin pathway genes	Positive	(Tamura et al., 2018)
PgNAC41-2	NAC	*Panax ginseng*	HMGR, SS, SE, DS, CYP716A47	Positive	(Liu et al.,2023b)
PgNAC72	NAC	*Panax ginseng*		Positive	
PnNAC03	NAC	*Panax notoginseng*	SS, SE, DS, PAL, C4H	Positive	(Zhang et al., 2025)
PgWRKY1	WRKY	*Panax ginseng*	DS, CYP716A47, CYP716A53v2, UGTs	Positive	(Nuruzzaman et al., 2016)
PgWRKY3	WRKY	*Panax ginseng*	CYP716A47, UGTs	Positive	
PgWRKY4X	WRKY	*Panax ginseng*	SE	Positive	(Yao et al., 2020)
PgWRKY8	WRKY	*Panax ginseng*	HMGR, FPS, DS, CYP716A53v2	Positive	(Xiu et al., 2016)
PqWRKY1	WRKY	*Panax quinquefolius*	Ginsenoside pathway	Positive	(Sun et al., 2013)
PgMYB2	MYB	*Panax ginseng*	DDS	Positive	(Liu et al., 2019)
PnMYB2	MYB	*Panax notoginseng*	Saponin pathway	Positive	(Xia et al., 2022)
PnMYB1	MYB	*Panax notoginseng*	Saponin pathway	Negative	(Man et al., 2023)
PnMYB4	MYB	*Panax notoginseng*	Saponin pathway	Negative	
PgERF120	AP2/ERF	*Panax ginseng*	Ginsenoside pathway	Positive	(Jiang et al., 2024)
PnERF1	AP2/ERF	*Panax notoginseng*	DS, SS, SE	Positive	(Deng et al., 2017)
PgGRAS68-01	GRAS	*Panax ginseng*	Ginsenoside pathway	Positive	(Liu et al.,2023a)
PgGT25-04	Trihelix	*Panax ginseng*	Ginsenoside pathway	Negative	(Zhu et al., 2023)

Epigenetic regulation adds an additional layer of control to saponin metabolism by modulating the accessibility of biosynthetic gene loci to transcriptional regulators. DNA methylation directly represses saponin biosynthetic genes, as demonstrated in *Eleutherococcus senticosus* under drought stress. Promoter demethylation of EsFPS, EsSS, and EsSE genes released transcriptional repression, allowing the binding of transcription factors such as EsMYB-r1 and subsequently activating saponin synthesis (S. Wang et al., 2024). Extending this regulatory paradigm, several NAC transcription factors in *E. senticosus* (EsJUB1, EsNAC047, EsNAC098, and EsNAC005) can target saponin biosynthetic gene promoters, but their binding is selectively inhibited by promoter DNA methylation, underscoring epigenetic gating as a key determinant of transcriptional control in saponin metabolism (Dong et al., 2024). Although specific histone modification studies on saponins are scarce, general principles suggest that active marks (e.g., H3K4me3) on saponin gene loci would favor expression under stress. Non-coding RNAs have also been implicated in the regulation of saponin metabolism. Genome-wide profiling in soapberry identified hundreds of miRNAs targeting both saponin biosynthetic genes and their upstream transcriptional regulators. Among these, miR5021, miR3630-3p, and miR858 are predicted to coordinately regulate multiple enzymes involved in triterpenoid saponin biosynthesis (Xu et al., 2023). Many of these miRNAs target TF families (bHLH, ERF, MYB, WRKY) that themselves control saponin metabolism (Xu et al., 2023). Emerging evidence points to a regulatory role for long non-coding RNAs (lncRNAs) in plant specialized metabolism. In Psammosilene, SA-elicited saponin production was associated with the expression of both biosynthetic genes and more than 4,600 candidate lncRNAs, indicating a likely lncRNA-mediated regulation of this pathway (Su et al., 2021).

Together, these transcriptional and epigenetic layers form an integrated regulatory system that confers both robustness and plasticity to saponin biosynthesis. This system enables plants to translate diverse environmental, hormonal, and developmental signals into precise metabolic outputs. Decoding these multilayered regulatory networks, particularly through single-cell multi-omics, chromatin profiling, and dynamic network modeling, will be essential for establishing predictive frameworks and rational engineering strategies aimed at fine-tuning saponin production in both plant and heterologous platforms.

### 4.4 Interplay between saponin and other secondary metabolite pathways

Saponin biosynthesis is integrated into the broader secondary metabolite network, with shared signals, precursors and transcriptional regulators often coordinating multiple pathways. Environmental or hormonal elicitors typically co-activate diverse metabolites. For example, salt stress in soybean simultaneously elevated alkaloids and saponins, indicating a common defense response (Zahra et al., 2022). In *Calendula*, JA elicitation not only enhanced saponin production but also reprogrammed sterol metabolism, marked by increased glycosylation of both triterpenoids (saponins) and sterols. This shift suggests an orchestrated redirection of metabolic flux from sterol biosynthesis toward the synthesis of defense-related saponins (Rogowska et al., 2022). Numerous TFs and miRNAs exhibit pleiotropic functions. This is illustrated in soapberry, where identified miRNA regulatory networks converge on TFs (such as bHLH, MYB, and ERF families) that govern saponin biosynthesis—many of these same TFs are known to regulate flavonoid or terpenoid pathways in other species (Xu et al., 2023). In summary, saponin metabolism is not strictly segregated but is often integrated with the biosynthesis of flavonoids, alkaloids, or other terpenoids in response to specific stimuli. Shared precursor pools derived from the isoprenoid and phenylpropanoid pathways, together with co-regulated transcription factor modules, support the view of an integrated metabolic network that coordinates defense-related processes with primary metabolism. Deciphering these cross-pathway feedback mechanisms will be essential for rational metabolic engineering, since enhancing saponin production may redirect flux and reprogram the broader terpenoid–phenylpropanoid network.

## 5 Synthetic biology and systems engineering of saponin biosynthesis

Recent advances in synthetic biology are fundamentally redefining how saponin biosynthesis is engineered, shifting the field from empirical pathway manipulation toward predictive, programmable, and system-level metabolic control. Rather than relying on static overexpression of individual genes, contemporary strategies emphasize dynamic regulation, quantitative flux balancing, and coordinated network optimization, reflecting the inherent complexity of plant metabolic systems.

The use of CRISPR-based transcriptional activation and repression enable precise modulation of endogenous biosynthetic genes, allowing quantitative tuning of key rate-limiting steps, including 3-hydroxy-3-methylglutaryl-CoA reductase, squalene synthase, and dammarenediol synthase (Lian et al., 2019). This regulatory mode more closely reflects native hormone-driven circuits, providing improved robustness and stability during prolonged cultivation. However, pathway flux remains constrained by low-efficiency cytochrome P450 monooxygenases and UDP-glycosyltransferases. Structure-guided engineering, directed evolution, and AI-assisted protein modeling, exemplified by AlphaFold, have markedly improved enzyme performance, enabling rational redesign of catalytic interfaces (Jumper et al., 2021). At the systems level, genome-scale metabolic modeling and flux balance analysis reveal quantitative trade-offs between sterol and saponin biosynthesis, guiding rational pathway reprogramming (Jang et al., 2022; Khan and Khan, 2026; Wang et al., 2025). Integration of these models into the Design–Build–Test–Learn framework enables iterative optimization across microbial and plant-based platforms, combining scalability with native glycosylation capacity (Lian et al., 2018).

Despite substantial progress, predictive control of saponin metabolism remains limited by incomplete understanding of regulatory interactions and intracellular compartmentalization. Future advances will rely on deeper integration of multi-omics, dynamic network modeling, and metabolic flux analysis to achieve fully programmable and robust biomanufacturing systems.

## 6 Translational strategies and industrial prospects for saponins

Collectively, these engineering strategies provide a foundation for rational pathway design and proof-of-concept demonstrations at the laboratory scale. The next critical step is to translate these advances into scalable production systems that can reliably supply these pharmaceutically valuable saponins. The convergence of synthetic biology, protein engineering, and bioprocess intensification is expected to transform rare saponins from laboratory curiosities into reliable raw materials for pharmaceuticals, nutraceuticals, and cosmetics (Lee et al., 2012). Establishing flexible production pipelines that combine plant- and microbe-based platforms with enzymatic upgrading will be key to achieving cost-effective, sustainable, and clinically relevant supplies of saponins.

**Figure 4:**
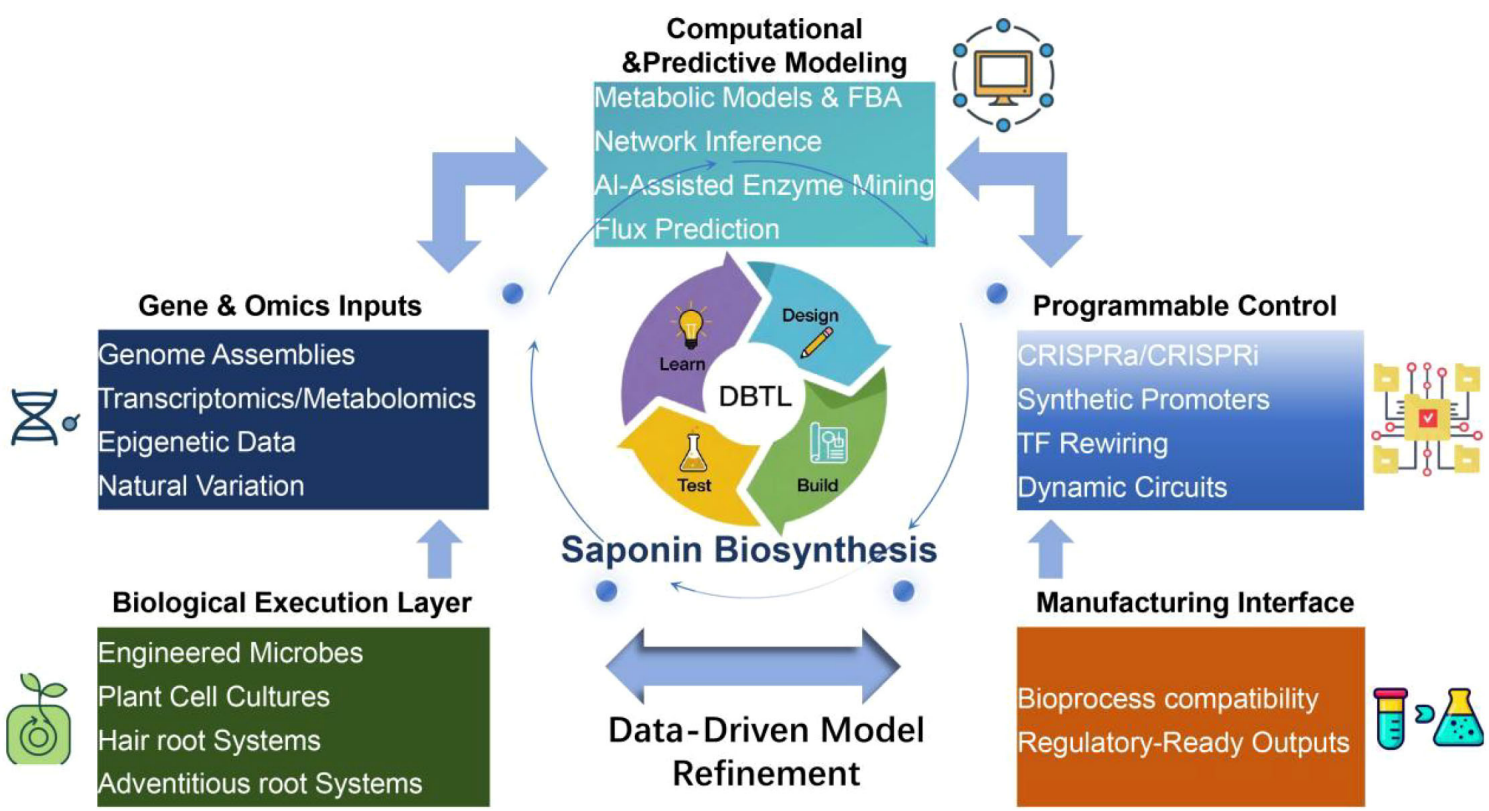
An iterative Design-Build-Test-Learn (DBTL) cycle for programmable saponin biomanufacturing. This framework integrates multi-omics data with computational modeling to predict pathway bottlenecks and regulatory nodes, which are then engineered using CRISPR-based tools and synthetic circuits. The resulting designs are implemented in diverse chassis (microbes, plant cell cultures) and iteratively optimized, culminating in scalable, industrial-grade production pipelines.

A conceptual Design–Build–Test–Learn (DBTL) framework (**Figure 4**) illustrates this programmable and predictive paradigm (Carbonell et al., 2018; Lawson et al., 2021). Multi-omicsinputs, including genome assemblies, transcriptomics, metabolomics, epigenetic information, and natural variation, are first integrated into computational and predictive modeling layers, where metabolic network inference, flux balance analysis, and AI-assisted enzyme mining enable quantitative prediction of pathway bottlenecks and regulatory leverage points (Bordbar et al., 2014; Chen et al., 2023). These predictions are translated into programmable biological interventions through CRISPRa/i-based gene modulation, synthetic promoter engineering, transcription factor rewiring, and dynamic regulatory circuits, enabling precise and tunable control of saponin biosynthetic flux (Lian et al., 2019). The resulting genetic designs are deployed across diverse biological chassis, ranging from microbial platforms to plant suspension cells, hairy roots, and adventitious root cultures, generating adaptable production systems tailored to specific saponin profiles. Experimental outputs continuously feed back into data-driven model refinement, closing the DBTL loop and improving predictive accuracy across iterative cycles (Carbonell et al., 2018; Lawson et al., 2021). Finally, a dedicated manufacturing interface ensures that optimized biosynthetic designs are compatible with scalable bioprocessing and regulatory requirements, thereby linking gene–enzyme–regulation layers to industrially deployable saponin biomanufacturing pipelines. Together, this framework illustrates how computational modeling, programmable gene regulation, and AI-assisted enzyme engineering can be unified into a closed-loop system that supports predictive, scalable, and regulatory-ready saponin biomanufacturing (**Figure 4**). As these technologies continue to mature, they are expected to redefine the boundaries of plant natural product engineering, providing a generalizable blueprint for the sustainable industrialization of complex specialized metabolites.

## 7 Concluding remarks

Saponins exemplify the intrinsic complexity of plant specialized metabolism, in which layered enzymatic modifications, transport processes, and dynamic regulatory networks collectively generate extraordinary chemical diversity. Advances in high-quality genome assemblies, multi-omics integration, and functional genomics have substantially clarified the enzymatic logic underlying scaffold formation, oxidation, and glycosylation, shifting the field from descriptive pathway elucidation toward mechanistic and systems-level understanding (Chen et al., 2023; Jo et al., 2025; Song et al., 2024). However, despite these achievements, regulatory control remains a principal bottleneck. Correlations between transcriptional reprogramming, epigenetic modifications, hormonal signaling, and saponin accumulation are frequently observed, yet causal relationships often remain unresolved, rendering most regulatory models probabilistic rather than deterministic. This regulatory uncertainty fundamentally constrains the predictive capacity of current engineering frameworks.

To overcome current limitations, emerging strategies increasingly integrate multi-omics diagnostics, computational modeling, and direct genetic intervention into unified engineering workflows. As illustrated in **Figure 4**, the Design–Build–Test–Learn (DBTL) paradigm provides a conceptual backbone for iterative refinement of biosynthetic systems, enabling the systematic identification of metabolic bottlenecks, rational pathway rewiring, and progressive enhancement of production performance (Carbonell et al., 2018; Lawson et al., 2021). In this context, AI-assisted enzyme engineering and genome-scale metabolic modeling are transforming pathway optimization from heuristic trial-and-error toward predictive and programmable design (Jang et al., 2022; Jumper et al., 2021; Khan and Khan, 2026). Nevertheless, the intrinsic complexity of plant metabolic networks, including extensive enzyme promiscuity, subcellular compartmentalization, and developmental heterogeneity, implies that fully deterministic control remains an aspirational goal rather than an immediate reality.

**Figure 5:**
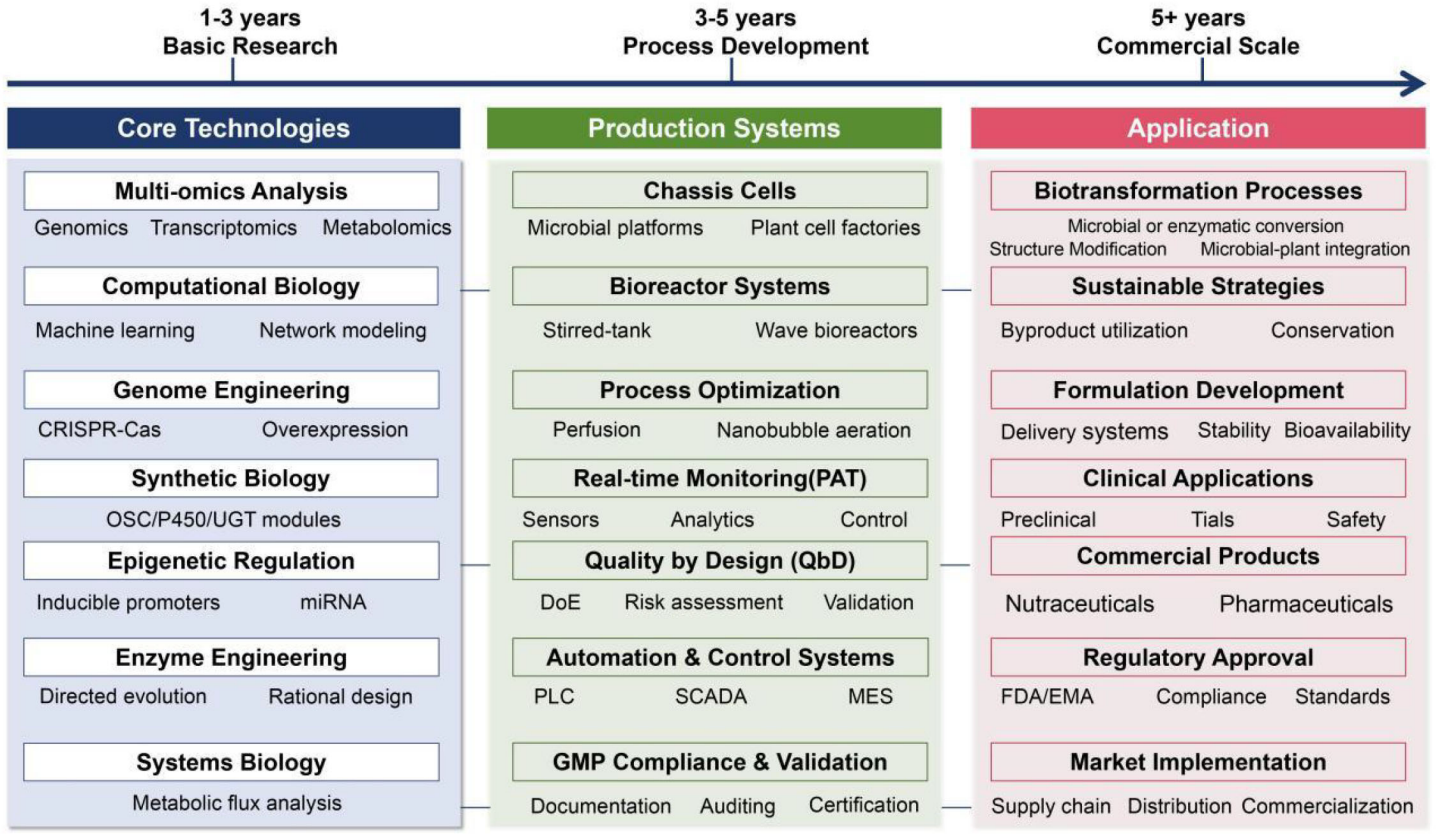
Roadmap for the industrial production and application of saponins. This diagram outlines the integrated pipeline from foundational technologies (multi-omics, synthetic biology) through scalable production systems (microbial platforms, plant cell cultures, bioprocess scale-up) to final applications in pharmaceuticals, functional foods, and clinical products. The workflow emphasizes the convergence of discovery, engineering, and translation to enable sustainable, high-value saponin manufacturing.

Beyond conceptual frameworks, the translation of these advances into scalable industrial processes represents the decisive frontier for saponin research. The **Figure 5** outlines an integrated roadmap for translational biomanufacturing, connecting foundational discovery, synthetic biology-enabled pathway engineering, chassis optimization, and bioprocess intensification into a continuous value chain. This framework highlights the necessity of coupling genetic design with fermentation engineering, downstream processing, and regulatory compliance, thereby bridging the long-standing gap between laboratory-scale demonstrations and industrial deployment.

Looking forward, the fusion of AI–driven metabolic modeling, high-throughput synthetic biology, and automated bioprocess control is expected to fundamentally reshape saponin biomanufacturing. Saponins are poised to serve not only as high-value natural products but also as a blueprint for engineering other complex plant secondary metabolites. These advances will accelerate the transition from empirical pathway manipulation toward truly programmable, predictive, and industrially scalable biosynthesis systems.
